# User Experience and Effects of an Individually Tailored Transdiagnostic Internet-Based and Mobile-Supported Intervention for Anxiety Disorders: Mixed-Methods Study

**DOI:** 10.2196/16450

**Published:** 2020-09-16

**Authors:** Kiona K Weisel, Anna-Carlotta Zarski, Thomas Berger, Tobias Krieger, Christian T Moser, Michael P Schaub, Dennis Görlich, Matthias Berking, David D Ebert

**Affiliations:** 1 Department of Clinical Psychology and Psychotherapy Friedrich-Alexander University Erlangen-Nürnberg Erlangen Germany; 2 Department of Clinical Psychology and Psychotherapy University of Bern Bern Switzerland; 3 Swiss Research Institute of Public Health and Addiction ISGF Associated to the University of Zurich Zurich Switzerland; 4 Institute of Biostatistics and Clinical Research Westfälische Wilhelms-Universität Münster Münster Germany; 5 Clinical, Neuro- & Development Psychology Vrije Universiteit Amsterdam Amsterdam Netherlands

**Keywords:** transdiagnostic, anxiety, depression, tailored, internet intervention

## Abstract

**Background:**

Internet interventions have been shown to be effective in treating anxiety disorders. Most interventions to date focus on single disorders and disregard potential comorbidities.

**Objective:**

The aim of this mixed-methods study was to investigate feasibility, user experience, and effects of a newly developed individually tailored transdiagnostic guided internet intervention for anxiety disorders.

**Methods:**

This study is an uncontrolled, within-group, baseline, postintervention pilot trial with an embedded qualitative and quantitative process and effect evaluation. In total, 49 adults with anxiety disorders (generalized anxiety disorder n=20, social phobia n=19, agoraphobia without panic n=12, panic with agoraphobia n=6, panic without agoraphobia n=4, subclinical depression n=41) received access to the 7-session intervention. We examined motivation and expectations, intervention use, user experience, impact, and modification requests. Qualitative data were assessed using semistructured interviews and analyzed by qualitative content analysis. Quantitative outcomes included symptom severity of anxiety and depression (Hamilton Anxiety Rating Scale [HAM-A], Quick Item Inventory of Depressive Symptomatology clinician rating [QIDS-C]), diagnostic status in clinical interviews (Mini International Neuropsychiatric Interview [MINI]), and web-based self-reports (Generalized Anxiety Disorder–7 [GAD-7], Center for Epidemiological Studies Depression Scale [CES-D], Beck Anxiety Inventory [BAI], Panic and Agoraphobia Scale [PAS], Social Phobia Scale [SPS], Patient Health Questionnaire–9 [PHQ-9]) at baseline and postassessment. Quantitative data was analyzed by comparing within-group means expressed as Cohen *d*.

**Results:**

Anxiety symptom severity (HAM-A *d*=1.19) and depressive symptoms (QIDS-C *d*=0.42) improved significantly, and 54% (21/39) no longer were diagnosed as having any anxiety disorder. The main positive effects were the general improvement of disease burden and attentiveness to feelings and risk situations while the main negative effects experienced were lack of change in disease burden and symptom deterioration. The most prevalent reasons for participation were the advantages of online treatment, symptom burden, and openness toward online treatment. Helpful factors included support, psychoeducation and practicing strategies in daily life; the main hindering factors were too little individualization and being overwhelmed by the content and pace.

**Conclusions:**

The intervention was found to be feasible and results show preliminary data indicating potential efficacy for improving anxiety and depression. The next step should be the evaluation within a randomized controlled trial. Concerning intervention development, it was found that future interventions should emphasize individualization even more in order to further improve the fit to individual characteristics, preferences, and needs.

## Introduction

Internet interventions can be effective means of treating mental health problems such as anxiety disorders [1-3]. Anxiety disorders are highly prevalent [4], and individuals suffering from anxiety disorders tend to experience significant impairment in quality of life and a decreased sense of well-being and occupational and family satisfaction [5,6]. Anxiety disorders have also been found to be highly comorbid and act as risk factors for developing other anxiety disorders (comorbidity rate of patients with generalized anxiety disorder in the past 12 months and any other anxiety disorder: 55.9%) or major depressive disorder (comorbidity of generalized anxiety disorder and major depressive disorder: 59.1%) [7-10].

The fact that the majority of individuals who suffer from a mental disorder do not receive treatment is a demanding public health issue [11]. One primary reason for nontreatment seeking behavior apart from structural barriers such as treatment availability is attitudinal barriers including preference for self-reliance, low perceived treatment need, poor mental health literacy, and fear of stigmatization [12-14]. Internet interventions offer many advantages that could help bridge this treatment gap. Meta-analytic evidence has found internet interventions to be efficacious with medium to large effect sizes for the treatment of anxiety disorders [3,15-17].

As anxiety disorders are often comorbid with other anxiety disorders and depression [7-9], there are advantages to treating all comorbid disorders within one transdiagnostic treatment protocol. Transdiagnostic treatment for anxiety disorders and depression can be applied to a broad range of patients regardless of their primary diagnosis as they are designed to target common underlying factors and also provide a variety of treatment [18,19]. Research investigating internet-based transdiagnostic treatment protocols for different anxiety disorders and comorbid depression has found these type of interventions to be efficacious [20-22].

Recently, there have been attempts to further individualize treatments according to the symptom profile and preferences of patients, which is also referred to as individual tailoring [23-25]. Beyond being able to address comorbidities, the main advantage is that patient preferences are considered in the treatment protocol, which could increase treatment motivation and therefore adherence and ideally also improve the outcome [26]. One trial found that effects in an internet-based intervention for depression were more pronounced when individual tailoring was applied compared with standardized treatment indicating the potential of individual tailoring [23]. To the best of our knowledge, no meta-analytic review exists on the sole effect of tailoring; however, meta-analytic evidence proposes that transdiagnostic and individual tailored approaches are promising when dealing with comorbidity with medium to large effect sizes for anxiety (*g*=0.82) and depression: (*g*=0.79) [27].

Some general aspects that remain unknown in internet interventions are (1) why individuals choose to participate, (2) how such interventions work including helpful and hindering factors [17,28,29], and (3) subjective impact including negative effects [30]. One way to explore these themes is through interviews with participants and qualitative data analysis.

The aim of this pilot feasibility study is to investigate a newly developed individually tailored transdiagnostic guided internet intervention for anxiety disorders with and without comorbid subclinical depression and explore feasibility. Qualitative and quantitative data and methods will be used to understand user experience focusing on motivation for participation and initial expectations, intervention use, and helpful and hindering factors. The impact of the intervention is explored through qualitative interviews as well as self-report and clinician-rated diagnostics on symptom severity, occurrence of clinical diagnoses, and positive and negative training effects. Finally, suggestions for intervention improvement and development will be derived.

## Methods

The paper describes the findings of a pilot feasibility study for a randomized controlled trial that was registered in the German Clinical Trials Register [DRKS00012656] and received ethical approval from Friedrich-Alexander University Erlangen-Nürnberg (144-16 B).

### Recruitment

Participants were recruited via German health insurance companies, a study webpage, and open recruitment strategies such as social media and Google Ads for a primary trial on the prevention of depression and anxiety [31]. Individuals with a clinical diagnosis of a major depressive disorder in the screening process were referred to another trial [32]. If they did not fulfill the criteria of the prevention trial due to a clinical diagnosis of an anxiety disorder and did not have a clinical diagnosis of major depressive disorder, they were referred to this study.

### Assessment of Eligibility

Participants were eligible to participate in the study if they fulfilled the following inclusion criteria of having a current diagnosis of an anxiety disorder (generalized anxiety disorder, panic disorder, agoraphobia, social phobia) assessed in the diagnostic interview based on the Mini International Neuropsychiatric Interview (MINI) [33] and signed informed consent without any of the following exclusion criteria: (1) history of psychosis, (2) bipolar disorder, (3) psychological treatment in the past 6 months, (4) currently on a waiting list for psychological treatment, (5) heightened suicidality, (6) having a current, or past 6 months, episode of a major depressive disorder. To increase internal validity, we decided not to include individuals with a major depressive disorder and redirected them to a different trial [32] as we assumed they might have other characteristics and needs.

### Study Design

**Figure 1:**
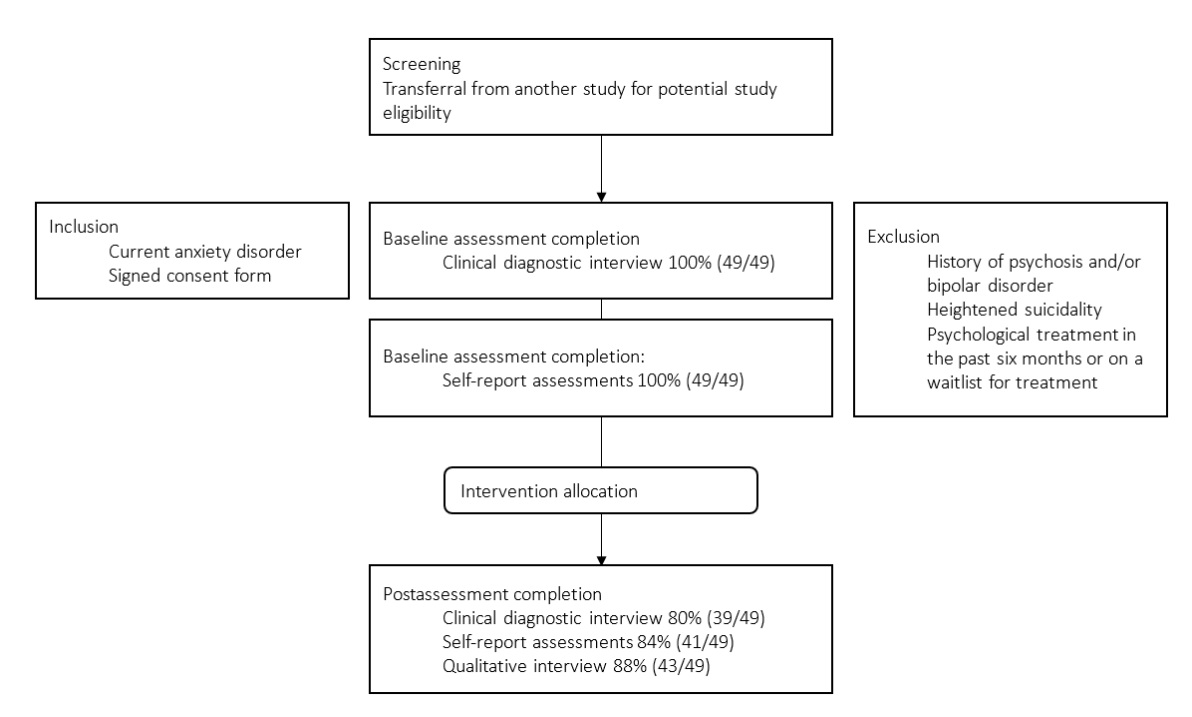
Study flow.

The study has an uncontrolled, within-group, baseline, postintervention design with an embedded qualitative and quantitative process evaluation. All participants (n=49) included in this study received access to the individually tailored transdiagnostic guided treatment for anxiety disorders. Clinical diagnostic interviews on diagnostic status and symptom severity of anxiety disorders and major depressive disorder were conducted at baseline and postassessment 8 weeks after intervention allocation. The participants also completed web-based self-report assessments on anxiety and depressive symptom severity and a question on treatment motivation and guidance preference at baseline and postassessment. A semistructured qualitative interview was conducted at postassessment. Figure 1 displays the study flow.

### Intervention

The internet intervention comprises 7 sessions plus one booster session. The content includes psychoeducation; methods to reduce incongruence between personal values, needs, and behavior; behavioral activation; exposure; and problem solving. The intervention is mainly text-based with additional elements such as short educational videos and audio files. To promote the transfer of acquired skills into daily life, participants could opt to receive short messages to their phone throughout the day (sent through an app or a messaging service) with motivational sentences or mini tasks referred to as Tiny Tasks. For more information on the reported intervention, see the published study protocol of the primary prevention trial [31]. See Textbox 1 for an overview of the intervention sessions.

**Session overview and elective modules.**

1. Behavioral activation: satisfying needs and goals

- Introduction to the training and core elements
- Strengthening motivation and personal goal setting
- Understanding the relationship between personal needs and values and identifying discrepancies
- Planning activities to strengthen core values

2. Behavioral activation: overcoming difficulties and pleasant activity scheduling

- Overcoming difficulties of behavioral activation
- Understanding the nature of avoidant behaviors
- Planning mood-enhancing activities

3. Psychoeducation

- Psychoeducational information on depression and anxiety including etiological and maintaining factors
- Identifying individual symptomatology and course of development

4. Cognitive restructuring

- Introduction to the causal relationship between cognitions and emotions
- Application of a thought record
- Identifying automatic negative thoughts and practicing cognitive flexibility

5. Exposure I or Problem solving I

- Practicing problem solving by distinguishing between solvable and unsolvable problems and applying a 6-step problem-solving plan
- Practicing exposition to fear-inducing situations based on a personal fear hierarchy

6. Exposure II or Problem solving II

7. Plan for the future

- Recap of the training
- Plan for the future and relapse prevention

8. Booster session

- Reflecting on goal attainment
- Further planning of the future

Elective modules: rumination and worries, acceptance, relaxation, reducing alcohol, self-worth, perfectionism, appreciation and gratitude, sleep

### Individual Tailoring

During the intervention development phase, there was an emphasis on individual tailoring which manifested itself through (1) tailoring to core clinical characteristics (any anxiety disorder or depression); (2) receiving optional Tiny Tasks; (3) choosing elective modules on various psychological topics such as acceptance, relaxation, or reducing alcohol based on interest, preference, and needs; (4) personal goal setting with monitoring of advancement in achieving goals and making adjustments throughout the intervention; (5) receiving personalized guidance by eCoaches who also monitored individual intervention use through adherence. The presented content in the intervention is triggered by patient input.

### Guidance and Adherence Monitoring

After completion of a session, patients receive written content-focused feedback by an eCoach. eCoaches are supervised psychologists or psychotherapists (in training) who provide manualized text-based feedback and monitor for adherence and potential crises throughout the intervention. In case of noncompliance to the intervention, eCoaches send reminder messages to encourage session completion. Patients are also sent automatic weekly email reminders by the platform in case of nonadherence.

### Assessments and Data Management

#### Qualitative Data

The interview manual was developed in collaboration with clinical experts and comprises 7 open questions including reasons for participation and expectations, training experience including helpful and hindering factors, impact of treatment, and modification requests. All participants were asked to participate in the qualitative interviews regardless of their actual treatment progress, intervention adherence, or session completion rates. Table 1 gives an overview of the topical domains and interview questions translated into English. The interviews were recorded and the content was transcribed verbatim.

**Table 1:** Overview of interview questions and domains.

Code	Domain	Question
Q1	Motivation for participation	Why did you participate in the online training?
Q2	Fulfilled expectations	Which expectations toward the training were fulfilled?
Q3	Unfulfilled expectations	Which expectations toward the training were not fulfilled?
Q4	Impact of online training	How has your disease burden changed by using the online training?
Q5	Helpful training event	What part of the training was particularly helpful in improving your psychological well-being?
Q6	Hindering training element	What would you have needed in addition from the training to help improve your psychological well-being?
Q7	Negative effects	Which elements of the training had no or negative effects on your psychological well-being?

#### Quantitative Assessments

Quantitative assessments took place during screening to complete study inclusion, at baseline before intervention access, and after intervention completion (8 weeks after baseline). Assessments comprised diagnostic interviews conducted by clinicians via telephone and web-based self-report assessments. The clinicians were blind to the fact that there was no control group. Figure 1 displays the study flow. The web-based assessments included measures of anxiety symptom severity, depression symptom severity, a question on treatment motivation, and guidance preference.

Anxiety disorders and major depressive disorder were assessed by an adaption of the MINI 5.0 [34]. Severity of anxiety symptoms was assessed by the Hamilton Anxiety Rating Scale (HAM-A; 14 items; α_T1_=.76) [35,36] and the Quick Item Inventory of Depressive Symptomatology clinician rating (QIDS-C; 16 Items; α_T1_=.64) [37,38] via telephone by diagnostic raters.

Generalized anxiety disorder and symptom severity were measured by the Generalized Anxiety Disorder 7 (GAD-7; 7 items; α_T1_=.82) [39,40]. The Beck Anxiety Inventory (BAI; 21 items; α_T1_=.91) was used to assess clinical anxiety [41]. Panic and agoraphobia symptoms were assessed by the Panic and Agoraphobia Scale (PAS; 13 items; α_T1_=.89) [42]. The Social Phobia Scale assessed social anxiety and pertains to fears of scrutiny during observations by others (SPS; 20 items; α_T1_=.93) [43,44].

Depressive symptoms were also assessed by the Patient Health Questionnaire (PHQ-9; 9 items; α_T1_=.73) [45] and the Center for Epidemiological Studies Depression Scale (CES-D; 20 items; α_T1_=.68) [46,47].

Motivation to receive online treatment as well as guidance preference were assessed by prompting participants to choose from a set of predefined answers in the web-based assessment. For an overview of all possible answers, see Multimedia Appendix 1.

#### Study Adherence

Adherence to study completion was monitored. To standardize the study adherence procedure, a systematic adherence protocol was instated. After noncompletion of an assessment or the diagnostic interview, participants were sent reminder emails after 7, 14, 21, and 28 days and text messages after 14, 21, and 28 days; reminder calls took place after 21 days. The text messages contained different motivational approaches to appeal to different mindsets such as helping others by providing data, having received the training in exchange for completing assessments, furthering scientific evidence, and supporting individuals of the study management team in completing their scientific degrees.

#### Quantitative Data Analysis

Feasibility of the intervention was assessed by exploring changes in the diagnostic status of any anxiety disorder or major depressive disorder and symptom improvement of anxiety and depression. Pre-post data were compared with paired *t* tests and expressed by Cohen *d* and the 95% confidence interval [48,49]. We also reported the mean percentage of symptom improvement per assessment scale. As this is a pilot feasibility trial exploring data and not testing hypotheses, we decided not to implement any strategies to estimate missing data and instead used completer data only. Due to the explorative nature of the trial, we also did not control the global significance level for the multiple testing problem. We also report baseline differences as median comparisons between self-report assessment completers and noncompleters investigated by the Mann-Whitney *U* test.

#### Qualitative Data Analysis

The recorded interviews were transcribed verbatim according to a predefined transcription guide. Content analysis and coding rules followed recommendations by Mayring [50]. The software program MAXQDA version 18.0.0 (VERBI GmbH) was used to analyze the qualitative data. Adherence to standards for reporting qualitative research was ensured by following the consolidated criteria for reporting qualitative research (COREQ) [51,52].

Taking an inductive approach, codes were developed by two researchers (KKW, LK) who used 50% of the raw data mindful of the topical domains. Codes were discussed until agreement was reached for a preliminary category system. Following this, the other 50% of raw data were analyzed by identifying codes and sorting them into the existing category system. If the codes did not match the existing system, the system was adapted by adjusting codes or creating new ones. The two researchers then discussed the coding and finalized the categorical system.

**Figure 2:**
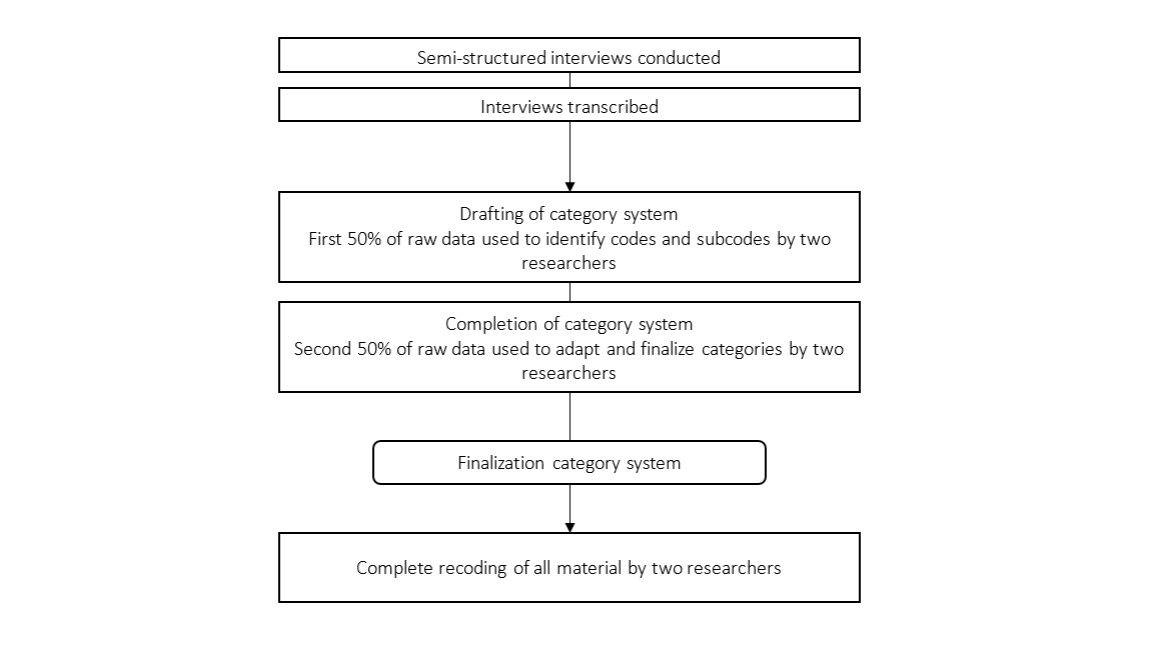
Study flow qualitative data analysis.

After finalization of the categorical system, two researchers (KKW, MNC) independently coded 10% of the data and analyzed their ratings in MAXQDA to determine an interrater agreement which is reported in the form of Cohen kappa (threshold was set to 10%). Figure 2 depicts the process of the qualitative data analysis.

## Results

### Baseline Characteristics

In total, 49 participants were included in the study, of which the majority was female (38/49, 78%). Participants were aged 40 years on average; the youngest participant aged 22 years and the oldest 68 years. Apart from two participants who resided in Switzerland and Austria, all others (47/49, 96%) had their residence in Germany. More than half of the participants (27/49, 55%) lived in cities with less than 100,000 inhabitants; 45% (22/49) of participants stated they lived in a city with more than 100,000 inhabitants. At baseline, all participants (49/49, 100%) included in the study had at least one anxiety disorder, while 18% (9/49) had at least two anxiety disorders and 6% (3/49) had three anxiety disorders. On average, participants had heightened symptoms of anxiety with a mean value of 21.29 (SD 7.79) on the HAM-A, 10.31 (SD 4.11) on the GAD-7, 38.35 (SD 10.96) on the BAI, 10.20 (SD 8.58) on the PAS, and 20.51 (SD 14.97) on the SPS. They also showed heightened symptom severity of depression with an average value of 8.92 (SD 4.41) on the QIDS-C, 21.71 (SD 6.58) on the CES-D, and 11.04 (SD 4.31) on the PHQ-9.

Of all participants, 43% (21/49) had no prior experience with psychotherapy and 57% (28/49) had some type of experience with psychotherapy. Of the 28 individuals with prior treatment experience, 32% (9/28) rated their experience as very helpful, 61% (17/28) found it somewhat helpful, and 7% (2/28) did not find their treatment to have been helpful. Considering prior experience with health-related trainings, 73% (36/49) claimed to have some and 27% (13/49) did not. See Table 2 for an overview.

**Table 2:** Baseline characteristics (n=49).

Characteristics		Value
**Sociodemographic characteristics**		
	Age in years, mean (SD)	40.45 (12.9)
	Gender, female, n (%)	38 (78)
**Country of residence, n (%)**		
	Germany	47 (96)
	Switzerland	1 (2)
	Austria	1 (2)
**Number of inhabitants, n (%)**		
	Less than 5000	8 (16)
	5000-10,000	5 (10)
	10,000-20,000	6 (12)
	20,000-50,000	3 (6)
	50,000-100,000	5 (10)
	100,000-500,000	8 (16)
	More than 500,000	14 (29)
**Ethnicity, n (%)**		
	White	45 (92)
	Other	4 (8)
**Education, n (%)**		
	8 years of schooling	1 (2)
	10 years of schooling	5 (10)
	Abitur or 3 to 3.5 year traineeship	19 (39)
	Bachelor or equivalent	8 (16)
	Masters or equivalent	15 (31)
	Doctorate degree	1 (2)
**Previous psychological treatment, n (%)**		
	Yes	28 (57)
	No	21 (43)
**For those who experienced psychological treatment (n=28): How helpful was it? n (%)**		
	Not helpful	2 (7)
	Somewhat helpful	17 (61)
	Very helpful	9 (32)
**Previous experience with health-related trainings, n (%)**		
	Yes	36 (73)
	No	13 (27)
**Symptom severity, mean (SD)**		
	HAM-A^a^ anxiety	21.29 (7.79)
	GAD-7^b^ anxiety	10.31 (4.11)
	BAI^c^ anxiety	38.35 (10.96)
	PAS^d^ anxiety	10.20 (8.58)
	SPS^e^ anxiety	20.51 (14.97)
	QIDS-C^f^ depression	8.92 (4.41)
	CES-D^g^ depression	21.71 (6.58)
	PHQ-9^h^ depression	11.04 (4.31)

^a^HAM-A: Hamilton Anxiety Rating Scale.

^b^GAD-7: Generalized Anxiety Disorder–7 item.

^c^BAI: Beck Anxiety Inventory.

^d^PAS: Panic and Agoraphobia Scale.

^e^SPS: Social Phobia Scale.

^f^QIDS-C: Quick Item Inventory of Depressive Symptomatology.

^g^CES-D: Center for Epidemiological Studies Depression Scale.

^h^PHQ-9: Patient Health Questionnaire–9 item.

### Assessment Completion

In total, 43 (43/49, 88%) qualitative interviews were conducted. The 6 individuals who did not complete the interview dropped out of the study, 2 of which informed the study team they did not want to continue and the other 4 could no longer be reached. The interview duration ranged from 1 minute 51 seconds to 8 minutes 21 seconds. The interrater agreement of codes in 10% of the interview data was 81% between raters (KKW, MNC).

As the quantitative analysis was based on assessment completer data and we had an assessment dropout of 16% (8/49), we additionally investigated baseline differences of symptom severity and intervention adherence rates of quantitative assessment completers and noncompleters (did not complete the web-based assessment at postintervention). Depression and anxiety symptom severity did not differ significantly between those who dropped out and those who completed the assessments. However, completers had significantly lower anxiety levels on the BAI (median 33) compared with noncompleters (median 44.50, *U*=80.5, *z*=–2.27, *P*=.02). For a complete overview of baseline differences, see Multimedia Appendix 1.

### Impact

Considering changes in diagnostic status at postassessment, of the individuals who completed the diagnostic interview, 46% (18/39) still had at least one anxiety disorder while 54% (21/39) no longer were diagnosed as having any anxiety disorder. There was a significant improvement of anxiety symptoms assessed by the HAM-A from 20.71 (SD 7.87, n=42) at baseline to 12.76 at postassessment (SD 9.18, n=42, T=7.0, df=41, *P*<.001, *d*=1.19, 95% CI 0.73 to 1.66), which translates to a mean symptom improvement of 38%. There was also a significant improvement of symptoms of depression assessed by the QIDS-C [38], from 8.4 (SD 4.13, n=42) to 6.38 at postassessment (SD 4.83, n=42, T=2.51, df=41, *P*=.016, *d*=0.42, 95% CI 0.01 to 0.86), which is a mean symptom of improvement of 24%.

All scales assessing anxiety symptom severity and depression symptom severity showed a significant improvement from baseline to postassessment. We also observed an improvement on the PAS [42] from baseline (mean 9.66 [SD 8.59], n=41) to postassessment (mean 8.12 [SD 7.06], n=41), which was not significant (T=1.86, df=40, *P*=.07, *d*=0.28, 95% CI –0.16 to 0.71). For a full overview see Tables 3 and 4.

**Table 3:** Completer baseline and postintervention data.

Questionnaire and assessment point		n^a^	mean (SD)	T score	df^b^	*P* value	*d*	95% CI	Mean symptom improvement (%)
**HAM-A^c^**				7.0	41	<.001	1.19	0.73 to 1.66	38.39
	T1	42	20.71 (7.87)						
	T2	42	12.76 (9.18)						
**GAD-7^d^**				4.94	40	<.001	0.75	0.31 to 1.20	29.85
	T1	41	10.05 (4.15)						
	T2	41	7.05 (3.97)						
**BAI^e^**				3.91	40	<.001	0.58	0.14 to 1.02	11.18
	T1	41	36.93 (10.54)						
	T2	41	32.80 (9.05)						
**PAS^f^**				1.86	40	.07	0.28	–0.16 to 0.71	15.94
	T1	41	9.66 (8.59)						
	T2	41	8.12 (7.06)						
**SPS^g^**				2.32	40	.03	0.35	–0.09 to 0.79	18.68
	T1	41	18.95 (13.73)						
	T2	41	15.41 (12.80)						
**QIDS-C^h^**				2.51	41	.02	0.42	–0.01 to 0.86	24.05
	T1	42	8.40 (4.13)						
	T2	42	6.38 (4.83)						
**CES-D^i^**				4.3	40	<.001	0.74	0.30 to 1.19	18.2
	T1	41	21.59 (6.42)						
	T2	41	17.66 (7.53)						
**PHQ-9^j^**				5.54	40	<.001	0.99	0.53 to 1.45	33.15
	T1	41	10.83 (3.92)						
	T2	41	7.24 (4.83)						

^a^completers only.

^b^df: degree of freedom.

^c^HAM-A: Hamilton Anxiety Rating Scale.

^d^GAD-7: Generalized Anxiety Disorder–7 item.

^e^BAI: Beck Anxiety Inventory.

^f^PAS: Panic and Agoraphobia Scale.

^g^SPS: Social Phobia Scale.

^h^QIDS-C: Quick Item Inventory of Depressive Symptomatology.

^i^CES-D: Center for Epidemiological Studies Depression Scale.

^j^PHQ-9: Patient Health Questionnaire–9 item.

**Table 4:** Diagnostic status depression and anxiety.

Clinical disorder assessed by the MINI^a^ and assessment point		Individuals with clinical diagnoses of valid responses n/N (%)
**Generalized anxiety disorder**		
	T1	20/49 (41)
	T2	6/39 (15)
**Social phobia**		
	T1	19/49 (39)
	T2	8/39 (21)
**Agoraphobia without panic**		
	T1	12/49 (24)
	T2	10/39 (26)
**Panic with agoraphobia**		
	T1	6/49 (12)
	T2	2/39 (5)
**Panic without agoraphobia**		
	T1	4/49 (8)
	T2	0/39 (0)
**Major depressive disorder**		
	T1	0/49 (0)
	T2	3/42 (7)
**Any anxiety disorder**		
	T1	49/49 (100)
	T2	18/39 (46)
**Two or more anxiety disorders**		
	T1	9/49 (18)
	T2	7/39 (18)
**Three or more anxiety disorder**		
	T1	3/49 (6)
	T2	1/39 (3)
**Subclinical depression** ^b^ **CES-D** ^c^ **≥16**		
	T1	41/49 (84)
	T2	20/41 (49)

^a^MINI: Mini International Neuropsychiatric Interview.

^b^Subclinical depression subgroup (CES-D ≥16) assessed by Center for Epidemiological Studies Depression Scale*.*

^c^CES-D: Center for Epidemiological Studies Depression Scale.

Any type of positive training effect was mentioned in 84% (36/43) of interviews while any type of negative training effect was identified in 30% (13/43). Only 2 interviews with negative effects had no mention of any positive effects; in one of these interviews, training discontinuation was mentioned.

Positive training effects stated were improvement of disease burden (n=26) including general improvement of disease burden (n=12), feeling of increased performance (n=3), improvement of depressive symptoms (n=2), less rumination (n=2), improvement of psychosomatic pain (n=1), reduction of suicidal and self-injurious thoughts (n=1), fewer panic attacks (n=1), less tension (n=1), more calmness (n=1), reduction of alcohol consumption (n=1), and improved sleep quality (n=1); attentiveness to feelings and risk situations (n=24); confrontation with one’s situation (n=20) including acceptance of oneself and others (n=8), focus on important areas of life (n=4), improvement of self-worth (n=2), knowing that one’s situation can change (n=2), preoccupation with oneself (n=1), proud of one’s achievements (n=1), and excited for future changes (n=1); insights and suggestions (n=12); more awareness for positivity and increased gratitude (n=6); and helpful entry to psychological treatment (n=1). Satisfaction with the online treatment was categorized as online treatment helpful (n=5), fulfilled expectations (n=6), excited about online treatment (n=6), and online treatment not helpful (n=4).

Negative training effects entailed lack of change in disease burden (n=11); symptom deterioration (n=9) including increased hopelessness (n=5), increased rumination (n=2), social withdrawal due to tension (n=1), general symptom deterioration (n=1); and training discontinuation (n=1).

### Intervention Use

In total, 98% (48/49) of participants completed the first session, and 65% (32/49) completed all 7 sessions. The booster session was completed by 55% (27/49). On average, participants took on average 9.44 (SD 3.78) weeks (range 4-18 weeks) to complete the intervention. See Table 5 for an overview.

**Table 5:** Completion rates.

Completion	Participants who completed session, n (%)	Participants who did not complete session, n (%)
Session 1	48 (98)	1 (2)
Session 2	44 (90)	5 (10)
Session 3	42 (86)	7 (14)
Session 4	40 (82)	9 (18)
Session 5	38 (78)	11 (22)
Session 6	33 (67)	16 (33)
Session 7	32 (65)	17 (35)
Booster session	27 (55)	22 (45)

In the noncompleter group, adherence intervention completion ranged from 0 to 3 sessions, while in the completer group, the average completion rate was mean 7.15 (SD 1.42) sessions.

Of the 38 participants who completed the fifth training session, 58% (22/38) chose to practice exposure to fear-inducing situations, while the other 42% (16/38) chose to practice problem-solving skills.

Regarding Tiny Tasks, only 10% (5/48) of individuals opted to not receive them, while 58% (28/48) chose to receive the light version with 3 daily reminders and motivational tasks and 31% (15/48) opted for the intense version with 5 messages per day.

### Motivation and Expectations

When asked in the online-based baseline assessment which type of guidance participants would like to receive, 74% (36/49) stated they would like guidance and feedback on completed training session, no one said they did not want guidance, and 27% (13/49) claimed they had no preference concerning guidance.

In the baseline assessment we asked the participants to select reasons, from predefined categories, why they wanted to participate in the online training. Almost all participants (47/49, 96%) selected the answer “I want to learn to cope with my complaints autonomously.” Approximately 67% (33/49) claimed that they found online training appealing and 31% (15/49) said that waiting times for psychotherapy are too long. For a complete overview see Multimedia Appendix 1.

There were 12 reasons associated with training motivation identified in the qualitative interviews: advantages of online treatment (n=38) including active self-help (n=29), time and place independent flexible use (n=4), anonymity and to not have to conduct face-to-face conversations (n=4), and something beyond self-help (n=1); symptom burden (n=29) including symptoms of anxiety and depression (n=18), not able to deal with one’s situation autonomously (n=5), unhappy with current life situation (n=2), sleep problems (n=2), loneliness (n=1), and feeling of putting burden on family (n=1); openness toward online treatment (n=12); desire for improvement (n=9); no expectations toward the online treatment (n=7); stressful life event (n=4); desire to better understand situation (n=3); negative psychotherapy experience (n=3); no face-to-face psychotherapy possible (n=3); heightened expectation of improvement by participation (n=3); interest in psychology and mental health (n=2); and positive experience with self-help (n=1). For a complete overview see Multimedia Appendix 1.

### Helpful and Hindering Factors

In total, 16 helpful factors and 10 hindering factors were identified. Of all interviews, 98% (42/43) had some mention of helpful factors and 74% (32/43) had some mention of hindering factors.

Helpful factors encompassed psychoeducation (n=14); support (n=20) including support by an eCoach (n=13), reminder emails (n=4), app notifications (n=2), and diagnostic interview (n=1); practice strategies in daily life (n=9); the structure of the program (n=8); relatable stories of testimonials (n=7); practicing thought protocol (n=7); planning activities (n=7); write down problems (n=7); confrontation with personal needs and values (n=7); elective modules (n=6); focus on personal situation (n=5); individual tailoring (n=4); neutral perspective on situations (n=3); problem solving (n=3); concrete instructions (n=2); and strategy collection (n=1). When individuals mentioned change but also stated that it could not directly be traced back to the training, it was categorized as other reasons for the change in disease burden (n=5).

Hindering factors comprised too little individualization of intervention (n=29) including too standardized (n=12), online treatment not sufficient (n=9), no feedback to specific inquiries (n=5), and too little personal contact (n=3); being overwhelmed by the content and pace (n=15); usability issues (n=8) including limited functionality of the app (n=6) and limited usability of weekly activity plan (n=2); difficulties doing exercises (n=7); motivational difficulties (n=6); difficulties to plan (n=3); not open to training elements (n=1); needs beyond the scope of the training (n=1); and stress (n=6).

### Modification Requests

There were 10 types of modification requests made, which were the desire for more intense support and more individualized feedback (n=10); longer treatment duration or more time to complete a module (n=9); exchange options with other participants (n=3); have limits of online treatment stressed (n=3); first aid plan (n=2); clearer structure of the activity plan (n=2); more printable content (n=2); more support to enhance motivation (n=2); more alternatives after having tried exercises (n=1); and be able to share content with friends and family (n=1).

## Discussion

### Principal Findings

The aim of this pilot feasibility study was to investigate an individually tailored transdiagnostic guided internet intervention for anxiety disorders with and without comorbid subclinical depression with an embedded qualitative and quantitative process evaluation.

Overall, the intervention was found to be feasible and results indicate potential efficacy concerning the improvement in anxiety and depression symptom severity. Moderate to large within effect sizes were found for anxiety and moderate effects for depression severity on all assessment scales apart from the PAS. Another finding in favor of the potential of the intervention is that while all individuals had an anxiety disorder at baseline, we found that the overall rate of anxiety disorders decreased by more than half. Positive effects stated by participants were general improvement of disease burden including improvement of anxiety and depression symptom severity and feeling equipped to deal with risk situations in the future. These findings are in line with previous work indicating that internet interventions are effective in treating anxiety disorders [1-3] and different anxiety disorders and comorbid depressive symptoms can be addressed transdiagnostically in one intervention [27,53-55].

Adherence to the intervention was found to be satisfactory with 65% of participants completing the intervention as intended, which lies just below some findings of adherence of internet-based guided self-help for anxiety disorders with 75% in a tailored group and 70.5% in a standardized treatment group [25]. The most prevalent reasons for participation found were advantages of the online treatment, symptom burden, and a general openness toward the online treatment. Participants found the most helpful factors to be the support provided, the psychoeducation, and being taught and encouraged to practice strategies in their daily routines.

Certain factors were perceived as hindering, such as the treatment not being individualized enough, at time they felt overwhelmed by the content and pace, and there were some usability issues. Some individuals struggled with motivation, regularly practicing, and integrating exercises into their daily lives, and others perceived stress outside of the intervention to be negative toward change. Many individuals also expressed the wish to have more contact with their eCoach going beyond the written messages and would have liked to have a personal conversation with their eCoach.

We also identified some negative effects. Although the quantitative data clearly showed an average improvement of anxiety and depression, the qualitative data revealed some negative effects such as an experienced lack of change in disease burden and symptom deterioration. Some individuals had heightened expectations of improvement prior to the treatment, which might be linked to greater disappointment and hopelessness if the treatment was perceived as ineffective. The negative effects were similar to what has been found in previous work, such as the occurrence of symptom deterioration or the emergence of novel symptoms [56]. When considering these negative effects, it is important to keep in mind that the individuals often mentioned negative and positive effects in one interview. This finding indicates that although many individuals improved in many different areas of life, it is possible that they did not improve in all the areas they would have liked to, it was not as effective as they expected, or they were not able or willing to put in the work to achieve the change they would have liked to experience. Although the number of negative effects mentioned was much less pronounced than positive treatment impact, this finding indicates the importance of exploring the use of methods beyond quantitative data such as qualitative data, as it can provide a more nuanced insight into user experience.

### Strengths and Limitations

This study has the following strengths: the combination of qualitative and quantitative methods; having a total of 43 independent voices included in the qualitative data analysis; conducting clinical interviews to assess diagnostic status; the high standard of conducting the qualitative analysis; the following of current recommendations in the field; and the high interrater reliability between coders.

This study also has some limitations. As this was an uncontrolled pilot feasibility study with an intervention group and no control group, there was an explorative analysis of only within pre-post data and there was no actual hypothesis testing; also, we did not apply any techniques to estimate missing data. The already small sample size was further decimated by study dropouts. Although we completed some statistical analyses of the quantitative data, the results should be interpreted with caution as the findings are based on a very small, self-selected, completer data sample. This should be kept in mind when regarding the quantitative findings. After investigating baseline symptom severity differences between assessment completers and noncompleters, we saw that noncompleters had higher symptom severity of anxiety on the BAI on average; therefore, it is possible that the effects based on completers only are an overestimation. Furthermore, individuals with a major depressive disorder at baseline were excluded from the study during the screening process to increase internal study validity. As participants self-selected to participate in the study, which is a typical occurrence in studies conducted in general population samples, this was an investigation of a highly selective population of individuals with an anxiety disorder, and findings from the qualitative data might not be generalizable to other populations. Also, the qualitative interview was completed after the intervention phase; therefore, questions on motivation and expectations were assessed in a retrospective manner and might be biased due to memory errors and time lapse. Last, it is possible that attrition rates are higher than they would be in a natural setting as the specificities of the study design such as diagnostic interviews might positively influence adherence.

### Learnings and Future Research

Our learnings for future studies when targeting individuals with anxiety disorders are that it is important to emphasize the self-help aspect of internet interventions, replace clinical and disorder-specific terms, highlight the flexible use options, and provide detailed information on what individuals can expect.

Although the intervention was heavily individualized and contained many elements of individual tailoring, participants still preferred to have the individualization options increased. First, future research should focus on the dose-response rate posing the question how flexible a treatment can be while still producing a positive outcome. Second, future research should investigate whether further individualization also increases initial acceptance and willingness to participate, which might increase the effects on a population level. Individual tailored interventions for anxiety and depression should also be systematically compared with standardized disorder specific treatment in one study.

Although not all individuals experienced negative effects, it still seems especially relevant to address negative effects due to their possible impact. If an individual who suffers from a mental disorder and has taken the step to seek treatment has a negative treatment experience, it could cause training discontinuation, detraction from seeking further psychological treatment, and chronification of symptomology, which is why it is important to intervene in a timely manner. One first step to address this would be to manage realistic expectations before and throughout the intervention, clarify possible limits of an online treatment, and engage or refer individuals after an intervention. To tackle issues of motivation and difficulties integrating exercises into daily life, it could be helpful to involve smartphones more often into the treatment protocol as they could function as an extension of the treatment into individuals’ private lives and are practical to deliver reminders. Future interventions should create a well-rounded support and guidance system that includes guidance adaptable in intensity combined with adherence and symptom monitoring.

There might also be other indicators of differential treatment outcome such as symptom severity, previous experience with mental health treatment, fear of stigma, and expectation of improvement, factors that could explain who benefits from internet interventions and who does not. For this reason, it is important to not only understand mediators of treatment but also investigate moderators of treatment and the combination of both. We also believe qualitative research should be used to understand why some individuals do not respond to treatment.

Concerning future qualitative research, it would be interesting to use machine learning techniques to analyze data. This could be done by analyzing content and words used by patients (eg, how someone speaks about themselves and their progress) as well as features of speech such as coherence, intonation, amplitude, pitch, and timbre. In addition to using these features to investigate user experience and impact, they might also be useful as an additional outcome assessment.

### Conclusion

The investigated individually tailored transdiagnostic guided internet intervention seems to be feasible and indicated potential to reduce anxiety and depression severity. The results suggest that when targeting individuals for this type of treatment, it can be helpful to emphasize the active self-help components in addition to the advantages of online treatments. The content should contain psychoeducation, emphasize practicing strategies in daily life, and be complemented by a support system that entails some type of guidance as well as adherence and symptom monitoring. Once thresholds for low adherence or heightened symptoms are crossed, mechanisms should be set in place to either adapt the intervention or guide individuals to further treatment. Further individualization of interventions should be explored to best adapt to patients’ characteristics, needs, and preferences.

## Appendix

Multimedia Appendix 1 Supplementary tables.

## Abbreviations

BAI: Beck Anxiety Inventory
CES-D: Center for Epidemiological Studies Depression Scale
COREQ: consolidated criteria for reporting qualitative research
GAD: Generalized Anxiety Disorder–7 item
HAM-A: Hamilton Anxiety Rating Scale
MINI: Mini International Neuropsychiatric Interview
PAS: Panic and Agoraphobia Scale
PHQ-9: Patient Health Questionnaire–9 item
QIDS-C: Quick Item Inventory of Depressive Symptomatology clinician rating
SPS: Social Phobia Scale
