# Supplementary material for: User Experience and Effects of an Individually Tailored Transdiagnostic Internet-Based and Mobile-Supported Intervention for Anxiety Disorders: Mixed-Methods Study
Source: J Med Internet Res. 2020 Sep 16;22(9):e16450. doi: 10.2196/16450 (PMC7527916; doi:10.2196/16450)
Supplement: Multimedia Appendix 1 [file jmir_v22i9e16450_app1.pdf]

# Multimedia Appendix 1

## User experience and effects of an individually tailored transdiagnostic internet-based and mobile-supported intervention for anxiety disorders: A mixed-methods study

*Kiona K. Weisel[1], M.Sc.; Anna-Carlotta Zarski[1], PhD; Thomas Berger[2], PhD; Tobias Krieger[2], PhD; Christian T. Moser[2], M.Sc.; Michael P. Schaub[3], PhD; Dennis Görlich[4], PhD; Matthias Berking[1], PhD; David D. Ebert[5], PhD*

*[1] Department of Clinical Psychology and Psychotherapy, Friedrich-Alexander University Erlangen-Nürnberg, Erlangen, Germany*

*[2] Department of Clinical Psychology and Psychotherapy, University of Bern, Bern, Switzerland*

*[3] Swiss Research Institute of Public Health and Addiction ISGF, Associated Institute of the University of Zurich, Zurich, Switzerland*

*[4] Westfälische Wilhelms-Universität Münster, Institute of Biostatistics and Clinical Research, Münster, Münster, Germany*

*[5] Clinical, Neuro- & Development Psychology, Vrije Universiteit Amsterdam, Amsterdam, Netherlands*

Corresponding author: Kiona K. Weisel, Friedrich-Alexander University Erlangen-Nürnberg, Nägelsbachstraße 25a, 91052 Erlangen, kiona.weisel@fau.de

**eTable 1.** Motivation for participation & guidance preference

| **What is your reason for participating in the online training?** | **n/N (%)** |
|---|---|
| I want to learn how to cope with my complaints autonomously. | 47/49 (95.9%) |
| I think an online training is appealing. | 33/49 (67.3%) |
| The waiting time for ambulant psychotherapy are too long. | 15/49 (30.6%) |
| I did not find other points of contact form my complaints. | 7/49 (14.3%) |
| I did not receive a spot for psychotherapy. | 2/49 (4.1%) |
| I do not have access to psychotherapy in my vicinity. | 1/49 (2.0%) |
| **Which type of guidance would you like to receive in the online training, if you could choose? (Your answer has no influence on what type of guidance you will actually receive during your participation in the online training.)** | |
| I would like to participate in the online training in a guided manner and receive feedback on completed training sessions. | 36/49 (73.5%) |
| I do not have a preference concerning the type of guidance in the online training. | 13/49 (26.5%) |
| I would like participate in the online training in an unguided manner without feedback on completed session modules. | 0/49 (0.0%) |

**eTable 2.** Overview over categorical system

| **Category 1** | **Category 2** | **Category 3** | **Category 4** | **Counts overall interviews** |
|---|---|---|---|---|
| **Motivation and expectations** | Symptom burden | Symptoms of anxiety and depression | | 18 |
| | | Not able to deal with situation autonomously | | 5 |
| | | Unhappy with current life situation | | 2 |
| | | Sleep problems | | 2 |
| | | Loneliness | | 1 |
| | | Feeling of putting burden on family | | 1 |
| | Advantages of online treatment | Active self-help | | 29 |
| | | Time and place independent flexible usage | | 4 |
| | | Anonymity and to not have to conduct face-to-face conversations | | 4 |
| | | Something beyond self-help | | 1 |
| | Openness towards online treatment | | | 12 |
| | Desire for improvement | | | 9 |
| | No expectations towards the online treatment | | | 7 |

| | | | | |
|---|---|---|---|---|
| | Stressful life event | | | 4 |
| | Desire to better understand situation | | | 3 |
| | Negative psychotherapy experience | | | 3 |
| | No face-to-face psychotherapy possible | | | 3 |
| | Heightened expectation of improvement by participation | | | 3 |
| | Interest in psychology & mental health | | | 2 |
| | Positive experience with self-help | | | 1 |
| **Training experience during the intervention** | | Psychoeducation | | 14 |
| | | Support | Support by an eCoach | 13 |
| | | | Reminder mails | 4 |
| | | | App notifications | 2 |
| | | | Diagnostic interview | 1 |
| | | Practice strategies in daily life | | 9 |
| | | Structure of the program | | 8 |
| | | Relatable stories of testimonials | | 7 |
| | | Practicing thought protocol | | 7 |
| | | Planning of activities | | 7 |
| | | Write down problems | | 7 |
| | | Confrontation with personal needs and values | | 7 |
| | | Elective modules | | 6 |
| | | Focus on personal situation | | 5 |
| | | Individual tailoring | | 4 |
| | | Neutral perspective on situations | | 3 |
| | | Problem solving | | 3 |
| | | Concrete instructions | | 2 |
| | Helpful factors | Strategy collection | | 1 |

| | | | | |
|---|---|---|---|---|
| | Other reasons for change in disease burden | | | 5 |
| | | Too little individualization of intervention | Too standardized | 12 |
| | | | Online treatment not sufficient | 9 |
| | | | No feedback to specific enquiries | 5 |
| | | | Too little personal contact | 3 |
| | | Being overwhelmed by the amount of content and pace | | 15 |
| | | Usability issues | Limited functionality of app | 6 |
| | | | Limited usability of weekly activity plan | 2 |
| | | Difficulties doing exercises | | 7 |
| | | Motivational difficulties | | 6 |
| | | Difficulties to plan | | 3 |
| | | Not open to training elements | | 1 |
| | | Needs beyond the scope of the training | | 1 |
| | Hindering factors | Stress | | 1 |
| **Modification requests** | | More intense support and more individualized feedback | | 10 |
| | | Longer treatment duration or more time to complete a module | | 9 |
| | | Exchange options with other participants | | 3 |
| | | Have limits of online treatment stressed | | 3 |
| | | First aid plan | | 2 |
| | | Clearer structure of the activity plan | | 2 |

| | | | | |
|---|---|---|---|---|
| | | More printable content | | 2 |
| | | More support to enhance motivation | | 2 |
| | | More alternatives after having tried out exercises | | 1 |
| | | Share content with friends and family | | 1 |
| **Impact** | Positive training effect - Impact of helpful factor | Improvement of disease burden | General improvement of disease burden | 12 |
| | | | Feeling of increased performance | 3 |
| | | | Improvement of depressive symptoms | 2 |
| | | | Less rumination | 2 |
| | | | Improvement of psychosomatic pain | 1 |
| | | | Reduction of suicidal and self-injurious thoughts | 1 |
| | | | Fewer panic attacks | 1 |
| | | | Less tension | 1 |
| | | | More calmness | 1 |
| | | | Reduction of alcohol consumption | 1 |
| | | | Improved sleep quality | 1 |
| | | Attentiveness to feelings and risk situation | | 24 |
| | | Confrontation with one's situation | Acceptance of oneself and others | 8 |
| | | | Focus on important areas of life | 4 |
| | | | Improvement of self-worth | 2 |
| | | | Knowing that one's situation can change | 2 |
| | | | Preoccupation with one self | 1 |

| | | | | |
|---|---|---|---|---|
| | | | Proud of one's achievements | 1 |
| | | | Excited for future changes | 1 |
| | | Insights and suggestions | | 12 |
| | | More awareness for positivity and increased gratitude | | 6 |
| | | Helpful entry to psychological treatment | | 1 |
| | Satisfaction with online treatment | Online treatment helpful | | 9 |
| | | Fulfilled expectations | | 6 |
| | | Excited about treatment | | 6 |
| | | Online treatment not helpful | | 4 |
| | Negative training effects - Impact of hindering events | Lack of change in disease burden | | 11 |
| | | Symptom deterioration | Increased hopelessness | 5 |
| | | | Increased rumination | 2 |
| | | | Social withdrawal due to tension | 1 |
| | | | General symptom deterioration | 1 |
| | | Training discontinuation | | 1 |

**eTable 3.** Comparison of baseline values completers versus non-completers

| | **Subgroup** | **N** | **Median** | **U** | **z** | **P** |
|---|---|---|---|---|---|---|
| Anxiety (HAM-A) | Completer | 42 | 21 | 125 | -1.06 | P=.30 |
| | Non-completer | 7 | 24 | | | |
| Anxiety (GAD-7) | Completer | 41 | 10 | 134.5 | -0.80 | P=.43 |
| | Non-completer | 8 | 10 | | | |
| Anxiety (BAI) | Completer | 41 | 33 | 80.5 | -2.27 | P=.02 |
| | Non-completer | 8 | 44.50 | | | |
| Anxiety | Completer | 41 | 9 | 124.5 | -1.07 | P=.29 |

| | | | | | | |
|---|---|---|---|---|---|---|
| (PAS) | Non-completer | 8 | 9 | | | |
| Anxiety (SPS) | Completer | 41 | 14 | 113.5 | -1.37 | P=.18 |
| | Non-completer | 8 | 24.50 | | | |
| Depression (QIDS-C) | Completer | 42 | 8 | 129.5 | -0.94 | P=.36 |
| | Non-completer | 7 | 9.50 | | | |
| Depression (CES-D) | Completer | 41 | 22 | 164 | 0.00 | P=1.0 |
| | Non-completer | 8 | 20.50 | | | |
| Depression (PHQ-9) | Completer | 41 | 10 | 150.5 | -0.37 | P=.72 |
| | Non-completer | 8 | 10.50 | | | |

*Notes*. HAM-A = Hamilton Anxiety Rating Scale. GAD-7 = Generalized Anxiety Disorder- 7 item, BAI = Beck Anxiety Inventory, PAS = Panic and Agoraphobia Scale, SPS = Social Phobia Scale, QIDS-C = Quick Item Inventory of Depressive Symptomatology, CES-D = Center for Epidemiological Studies Depression Scale, PHQ-9 = Patient Health Questionnaire-9 item
